# An Intrasheath Separation Technique for Nerve-Sparing High Ligation of the Inferior Mesenteric Artery in Colorectal Cancer Surgery

**DOI:** 10.3389/fonc.2021.694059

**Published:** 2021-06-24

**Authors:** Zhifang Zheng, Xiaojie Wang, Ying Huang, Xingrong Lu, Xiaozhen Zhao, Pan Chi

**Affiliations:** ^1^ Department of Colorectal Surgery, Fujian Medical University Union Hospital, Fuzhou, China; ^2^ Department of General Surgery, Fujian Medical University Union Hospital, Fuzhou, China; ^3^ Laboratory of Clinical Applied Anatomy, Fujian Medical University, Fuzhou, China

**Keywords:** colorectal cancer, high ligation, inferior mesenteric artery (IMA), inferior mesenteric plexus, vascular sheath

## Abstract

**Purpose:**

To investigate the relationship between the left trunk of the inferior mesenteric plexus (IMP) and the vascular sheath of the inferior mesenteric artery (IMA) and to explore anatomical evidence for autonomic nerve preservation during high ligation of the IMA in colorectal cancer surgery.

**Methods:**

We evaluated the relationship in 23 consecutive cases of laparoscopic or robotic colorectal surgery with high ligation of the IMA at our institute. Anatomical dissection was performed on 5 formalin-fixed abdominal specimens. A novel anatomical evidence-based operative technique was proposed.

**Results:**

Anatomical observation showed that the left trunk of the IMP was closely connected with the IMA and was involved in the composition of the vascular sheath. Based on anatomical evidence, we present a novel operative technique for nerve-sparing high ligation of the IMA that was successfully performed in 45 colorectal cancer surgeries with no intraoperative complications and satisfactory postoperative urogenital functional outcomes.

**Conclusion:**

The left trunk of the IMP is involved in the composition of the IMA vascular sheath. This novel anatomical evidence-based operative technique for nerve-sparing high ligation of the IMA is technically safe and feasible.

## Introduction

Ligation of the inferior mesenteric artery (IMA) is a key procedure during surgery for left colon and rectal cancer. Ligation of the IMA from the origin of the aorta is defined as high ligation, and ligation below the origin of the left colic artery is defined as low ligation (1). Based on oncological, technical, and anatomical considerations, the location of IMA ligation is still controversial. Although there is no consistent evidence that high ligation of the IMA has a survival benefit (2–4), it may improve the lymph node dissection rate (5) and tumor staging accuracy (3). Although high ligation of the IMA results in a decreased blood supply to the distal colon (6, 7), it simultaneously contributes to low anastomosis with no tension during low anterior resection for rectal cancer (2, 8). In fact, there is no significant difference in the incidence of anastomotic leakage between high and low ligation of the IMA (9, 10). With the popularity of laparoscopic surgery, laparoscopic high ligation of the IMA is much easier than low ligation and is not associated with a prolonged operation or an increase in blood loss (11). Therefore, high ligation of the IMA is still preferred by most surgeons in colorectal cancer surgery.

**Figure 1:**
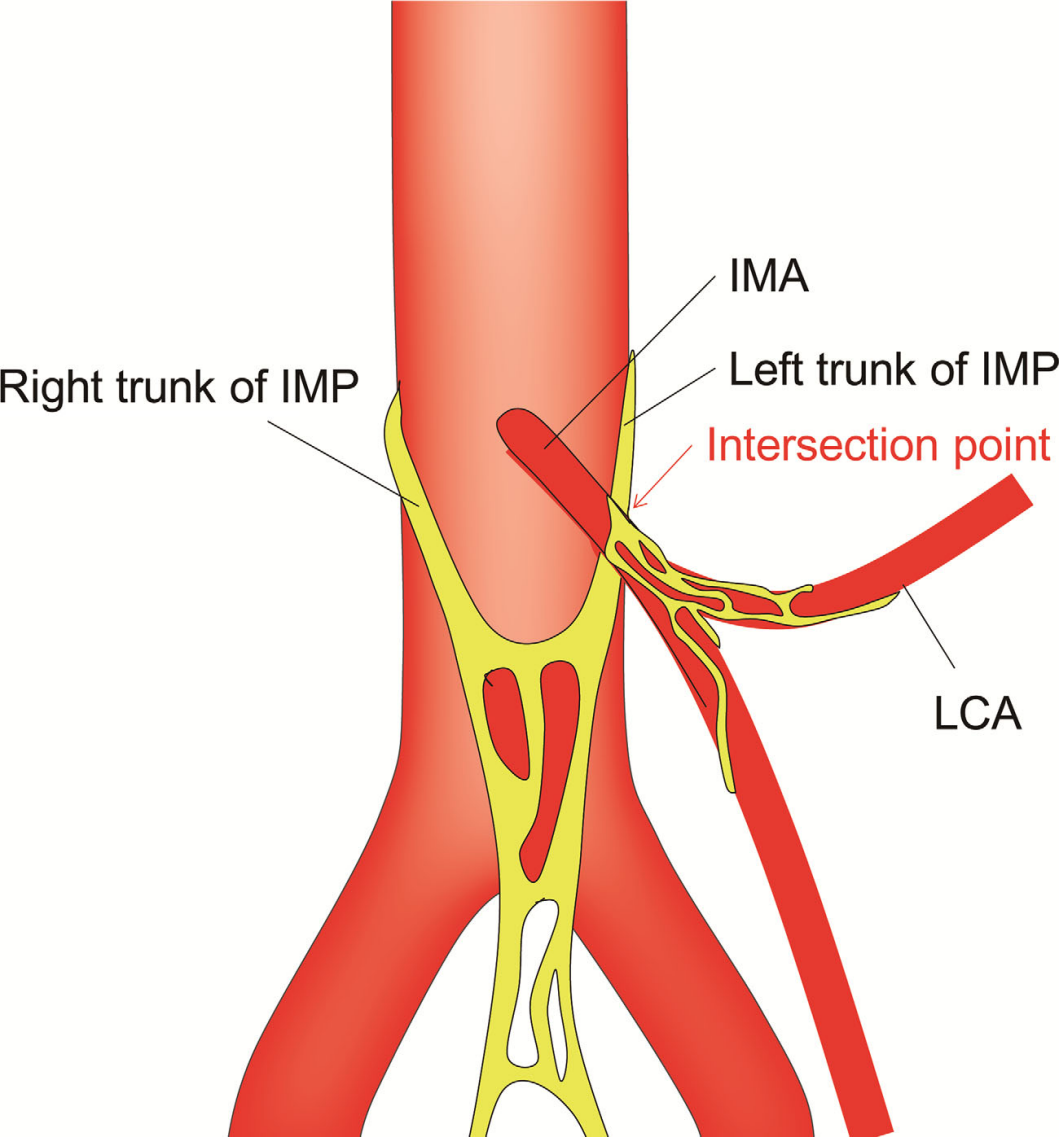
Schematic representation of the relationship between the IMA and the surrounding autonomic nerve plexuses. IMP, inferior mesenteric plexus; IMA, inferior mesenteric artery; LCA, left colic artery.

However, it is widely accepted that high ligation of the IMA carries a risk of damage to the surrounding autonomic nerve plexuses (12, 13), which may lead to postoperative urogenital dysfunction (14). The reason is that the bilateral trunks of the inferior mesenteric plexus (IMP) pass through the root of the IMA. At present, anatomical studies of the relationship between the root of the IMA and the autonomic nerve plexus are very limited. Previous studies (15–17) have shown that the right trunk of the IMP is located relatively far from the root of the IMA and does not cross the root of the IMA, while the left trunk of the IMP crosses over the IMA (**Figure 1**). Therefore, high ligation of the IMA may be more likely to cause damage to the left trunk of the IMP. It is well known that the IMA is wrapped in a vascular sheath. Unfortunately, the anatomical relationship between the left trunk of the IMP and the IMA vascular sheath has not been reported.

Therefore, the present study aimed to investigate the anatomical relationship between the left trunk of the IMP and the IMA vascular sheath, providing anatomical evidence for autonomic nerve preservation in high ligation of the IMA in colorectal cancer surgery.

## Materials and Methods

### Samples

To investigate the relationship between the IMP and the IMA vascular sheath, we prospectively collected clinicopathological data and surgical videos of 23 consecutive cases of laparoscopic or robotic colorectal surgery with high ligation of the IMA at our institution in October 2019. Five formalin-fixed cadavers (3 males and 2 females; mean age, 72.3 years) donated to Fujian Medical University were dissected with the assistance of binocular loupes. Cadavers with a deformed anatomy in the region of the IMA resulting from a previous abdominal surgery were excluded.

From January 2020 to February 2020, based on the anatomical results, a novel operative technique for nerve-sparing high ligation of the IMA was performed in laparoscopic or robotic surgery for 45 patients with advanced colorectal cancer by an experienced colorectal surgeon (Prof. Pan Chi) at the Fujian Medical University Union Hospital. The reliability of the anatomical evidence and the key points of the surgical techniques were evaluated. The operative procedures were recorded on video. Informed consent was obtained from all participating patients. This study was approved by the Institutional Review Board of the Fujian Medical University Union Hospital (no. 2020KY092).

### Definition of the IMA Sheath

The IMA vascular sheath was defined as the tissue located between the surface of the IMA adventitia and the collagenous layers isolated from adipose tissue coupled with the surrounding connective tissue, including layers of the autonomic nerve plexus, adipose tissue, collagenous fibers, and microvessels (18, 19).

### Urogenital Function

The patients’ urinary functional status was evaluated by the International Prostate Symptom Score (IPSS). Erectile function was evaluated by the 5-item version of the International Erectile Function Index Questionnaire (IIEF-5) (20). The total IIEF-5 score ranged from 1 to 25, with a lower score indicating more severe erectile dysfunction.

### Statistical Analysis

No statistical analysis was necessary.

## Results

### Anatomical Observations

We observed and evaluated the relationship between the IMP and the IMA vascular sheath in 23 patients who underwent laparoscopic or robotic colorectal surgery with high ligation of the IMA. The mean age of the enrolled patients was 58.2 ± 9.2 years old, with a male:female ratio of 1.5:1. The tumors were located in the left colon (n=3), sigmoid colon (n=5), and rectum (n=15). Regarding the stage classification, 5, 6, and 12 patients had stage I, II, and III disease, respectively. Among them, 8 patients received preoperative neoadjuvant therapy.

**Figure 2:**
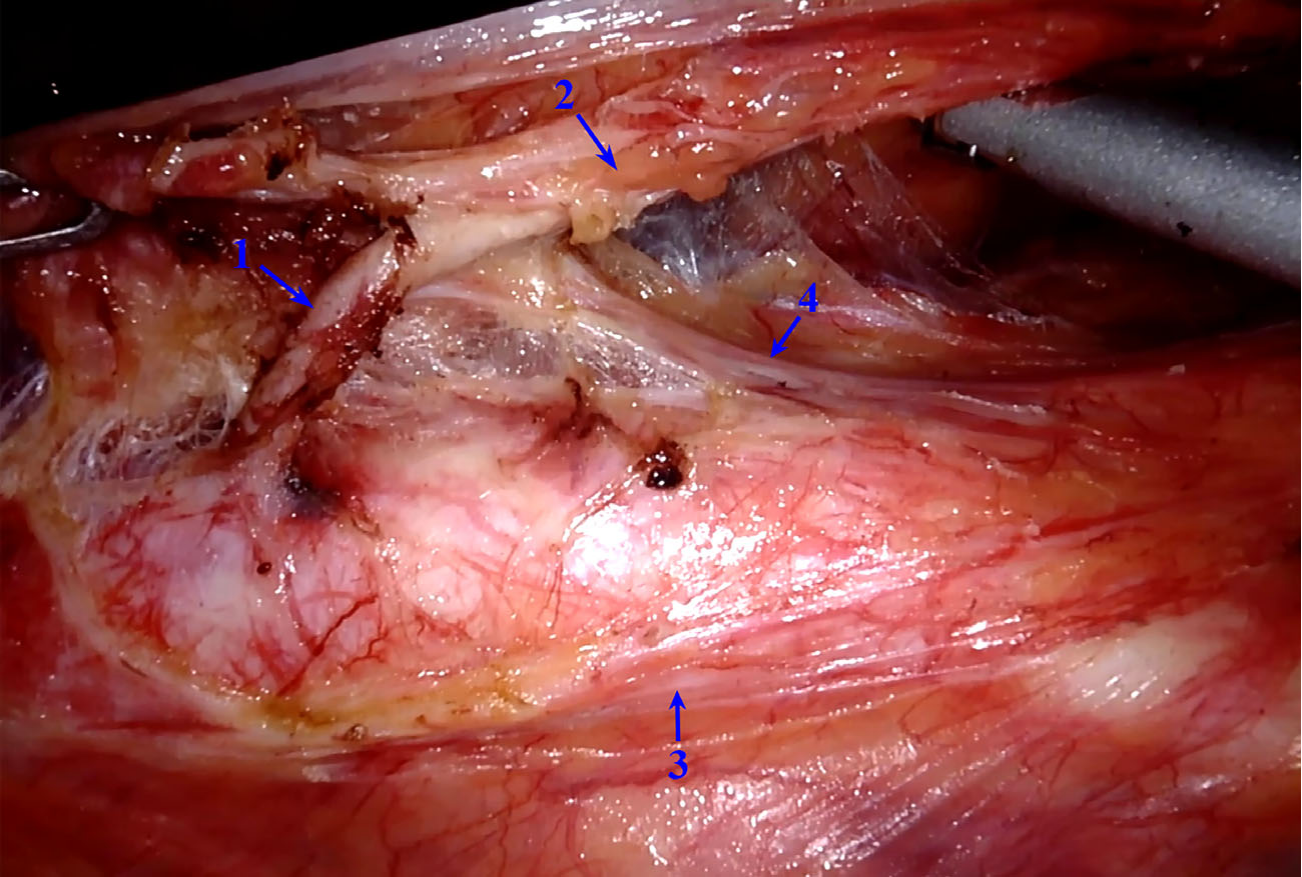
Intraoperative image of the relationship between the IMA and IMP. The left trunk of the IMP is tightly connected to the IMA, forming part of the IMA vascular sheath. IMP, inferior mesenteric plexus; IMA, inferior mesenteric artery. 1, IMA without the vascular sheath; 2, IMA with the vascular sheath; 3, right trunk of the IMP; 4, left trunk of the IMP.

**Figure 3:**
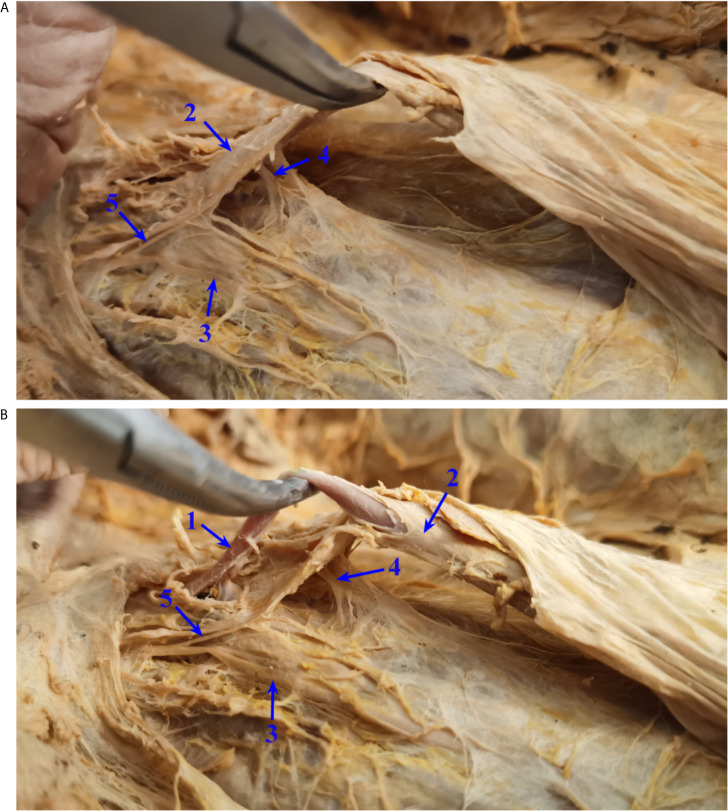
Relationship between the IMA and IMP in formalin-fixed cadavers. **(A)** The left trunk of the IMP could not be removed from the IMA sheath in formalin-fixed cadavers. **(B)** The left trunk of the IMP could be separated from the IMA only when the IMA sheath was peeled off. IMP, inferior mesenteric plexus; IMA, inferior mesenteric artery. 1, IMA without the vascular sheath; 2, IMA with the vascular sheath; 3, right trunk of the IMP; 4, left trunk of the IMP; 5, branches from the main right trunk of the IMP to the left colon.

In all 23 patients, typical structural relationships were observed, as follows: the right trunk of the IMP was located relatively far from the root of the IMA and did not cross the root of the IMA, while the left trunk of the IMP was closely connected with the IMA and was involved in the formation of the IMA vascular sheath. The nerve fibers of the left trunk of the IMP could be raised with the IMA by pulling the IMA upward during the operation, similar to a “tent” (**Figure 2**). The anatomical relationships were further validated in formalin-fixed cadavers. The main right trunk of the IMP was located relatively far from the root of the IMA, while several tiny branches supplying the left colon along the IMA were observed (**Figure 3A**). The left trunk of the IMP could not be removed from the IMA sheath in the formalin-fixed cadavers (**Figure 3A**). The left trunk of the IMP could be separated from the IMA only when the IMA sheath was peeled off (**Figure 3B**).

### Anatomical Evidence-Based Surgical Technique

**Figure 4:**
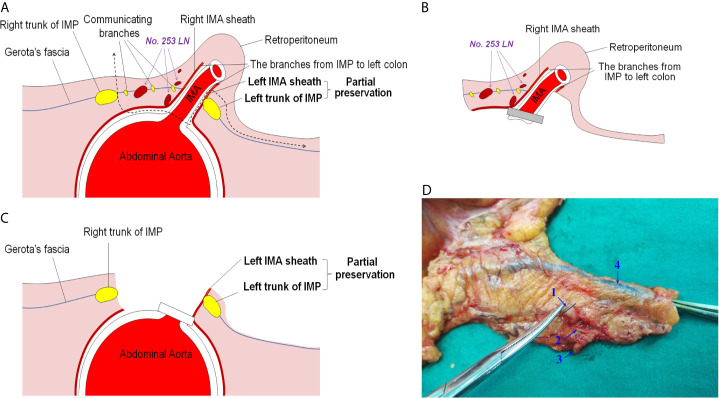
Illustration of the novel operative technique for nerve-sparing high ligation of the IMA. **(A)** Schematic diagram of the novel operative technique: intrasheath separation of the IMA and partial preservation of the left IMA sheath along with the left trunk of the IMP. **(B)** Diagram of a portion that had been removed intraoperatively. **(C)** Diagram of a preserved part intraoperatively. **(D)** Representative specimen after application of the novel operative technique. IMA, inferior mesenteric artery; IMP, inferior mesenteric plexus. 1, IMA; 2, right IMA sheath; 3, enlarged lymph node; 4, inferior mesenteric vein.

**Figure 5:**
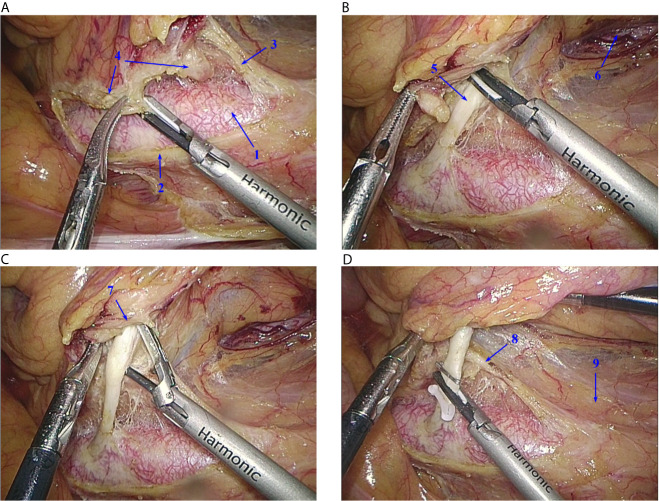
Operative technique. **(A)** Lymph nodes around the root of the IMA are dissected in the nerve-free “window”, and the root of the IMA is exposed. **(B)** From the right side of the root of the IMA, the vascular sheath is peeled off. **(C)** The left side of the IMA vascular sheath is preserved. **(D)** The IMA is ligated and cut at its root. IMA, inferior mesenteric artery; IMP, inferior mesenteric plexus. 1, Abdominal aorta; 2, right trunk of the IMP; 3, left trunk of the IMP; 4, lymph nodes; 5, IMA; 6, left ureter; 7, right vascular sheath of the IMA; 8, left vascular sheath of the IMA; 9, Gerota’s fascia.

Based on the above anatomical evidence, we proposed a novel operative technique for nerve-sparing high ligation of the IMA called intrasheath separation of the IMA and partial preservation of the left IMA sheath along with the left trunk of the IMP (**Figures 4A–C**). The details of the surgical procedures are shown in the attached **video**. Briefly, the dissection commenced above the junction point of the bilateral trunks of the IMP with an incision in Gerota’s fascia and exposure of the abdominal aorta. Along the surface of the abdominal aorta and the medial side of the right trunk of the IMP, the root of the IMA was exposed, and the no. 253 lymph nodes were dissected at the root of the IMA (**Figure 5A**). Subsequently, the right IMA vascular sheath was cut at the root of the IMA and peeled off slowly upward along the IMA (**Figure 5B**). Approximately 1.5 cm away from the root of the IMA, the left trunk of the IMP was cross-fused with the IMA vascular sheath. Starting from the root of the IMA, the left IMA vascular sheath was separated at the space between the left wall of the IMA and the left IMA vascular sheath until after the left trunk of the IMP had passed, and then the left IMA vascular sheath along with the left trunk of the IMP could be preserved (**Figure 5C**). Finally, the IMA was ligated and cut at its root (**Figure 5D**). **Figure 4D** shows representative postoperative specimens containing the right IMA vascular sheath, while the left IMA vascular sheath and the left trunk of the IMP were preserved. Several enlarged lymph nodes were visible in this specimen and were confirmed as metastatic lymph nodes by postoperative pathology.

### Surgical Outcomes

The novel anatomical evidence-based operative technique for nerve-sparing high ligation of the IMA was successfully performed in 45 consecutive patients who underwent laparoscopic or robotic surgery for colorectal cancer. The clinicopathological data of the patients are presented in **Table 1**. The bilateral trunks of the IMP could be identified and well protected during the operation. The mean operative time from exposure of the abdominal aorta to high ligation of the IMA was 8.2 ± 2.1 min (range, 6-11 min). The mean blood loss during high ligation of the IMA was 5 ± 6.2 ml (range, 0-20 ml), without intraoperative complications. The preoperative IPSS and IIEF-5 score was 3.1 ± 2.1 and 18.7 ± 5.6, respectively, and the postoperative urogenital function was satisfactory (IPSS at 6 months postoperatively: 4.9 ± 3.6; IIEF-5 score at 12 months postoperatively: 17.1 ± 5.1).

**Table 1:** Clinical and pathological characteristics of participating patients.

Variable	Value
No. of cases	45
Age, mean (SD), years	62.0 ± 10.3
Sex (male/female)	30/15
Body mass index, mean (SD), kg/m^2^	23.2 ± 2.6
Tumor site, n (%)	
Left colon	5 (11.1)
Sigmoid colon	9 (20.0)
Rectum	31 (68.9)
Differentiation, n (%)	
Well or moderate	39 (86.7)
Poor, mucinous or signet-ring cell	6 (13.3)
Neoadjuvant chemoradiotherapy, n (%)	
Yes	15 (33.3)
No	30 (66.7)
pTNM stage, n (%)	
I	11 (24.4)
II	14 (31.2)
III	20 (44.4)
Lymph nodes retrieved, mean (SD)	16.1 ± 8.9
Positive lymph nodes, mean (SD)	0.9 ± 1.3
Positive no. 253 lymph nodes, n (%)	3 (6.7)
Total operative time, mean (SD), min	185 ± 35.7
Operative time from exposure of the abdominal aorta to high ligation of the IMA, mean (SD), min	8.2 ± 2.1
Estimated blood loss, mean (SD), ml	78.9 ± 31.4
Estimated blood loss during high ligation of the IMA, mean (SD), ml	5 ± 6.2
IPSS	
Preoperatively	3.1 ± 2.1
Six months postoperatively	4.9 ± 3.6
IIEF-5 score	
Preoperatively	18.7 ± 5.6
Twelve months postoperatively	17.1 ± 5.1

SD, standard deviation; IMA, inferior mesenteric artery; IPSS, International Prostate Symptom Score; IIEF-5, International Index of Erectile Function 5.

## Discussion

With the improvement of oncological results (21), the quality of life of patients after colorectal cancer surgery has become increasingly important, and autonomic nerve preservation has gradually attracted the attention of surgeons and scholars (22). The root of the IMA is widely considered to be one of the critical areas for autonomic nerve damage during surgery (12, 13). However, anatomical studies on the relationship between the roots of the IMA and the surrounding autonomic nerves are limited, and there are still few reports on autonomic nerve-preserving surgical techniques for high ligation of the IMA based on reliable anatomical evidence. Therefore, the present study explored the relationship between the IMP (especially the left trunk of the IMP) and the IMA by anatomical observation. It was found that the left trunk of the IMP formed part of the IMA vascular sheath, and guided by anatomical evidence, a novel surgical technique for nerve-sparing high ligation of the IMA was proposed.

In this study, it was found that the main right trunk of the IMP did not cross the root of the IMA, and the left trunk of the IMP crossed over the IMA, which is consistent with previous studies (15–17). In contrast, previous anatomical studies of cadavers have neglected the existence of vascular sheaths. Intraoperatively, we found that the left trunk of the IMP formed part of the IMA vascular sheath, which was confirmed in formalin-fixed cadavers. This anatomical finding is important because it means that extrasheath separation of the IMA will inevitably cause damage to the left trunk of the IMP in the case of high ligation of the IMA, which may lead to postoperative urogenital dysfunction.

To protect autonomic nerves during surgery, several studies have presented different opinions. The results from a randomized controlled trial [HIGHLOW trial (23)] showed that low ligation of the IMA in laparoscopic anterior resection for rectal cancer reduced genitourinary dysfunction. Many studies have found that IMA lymph node metastasis is an important prognostic factor in patients with colorectal cancer (24–26). In the Japanese Society for Cancer of the Colon and Rectum (JSCCR) guidelines (27), lymph node dissection of the root of the IMA, namely, D3 lymph node dissection, is recommended for patients with preoperative clinical stage T2 disease or higher and rectal cancer with lymph node metastasis. The American Society of Colon and Rectal Surgeons Clinical Practice Guidelines (28) recommend that patients suspected of having IMA lymph node metastasis should undergo high ligation and lymph node dissection of the root of the IMA. Therefore, low ligation of the IMA may not be appropriate in this subset of patients. Yang et al. (17) suggested that high ligation of the IMA should be performed at a distance distal to the intersection of the left trunk of the IMP and the IMA to protect the left trunk of the IMP. However, whether the no. 253 lymph nodes can be dissected completely by the above method and the subsequent oncological results still need further verification. Liang et al. (14) believed that the left trunk of the IMP should be adequately cleared from “behind” the IMA during surgery, but it was not separated *via* the intrasheath method, which would inevitably cause damage to the left trunk of the IMP to some extent. To maintain a balance between oncology and function, based on the anatomical findings of the present study, we proposed a novel surgical technique for nerve-sparing high ligation of the IMA called intrasheath separation of the IMA and partial preservation of the left IMA sheath along with the left trunk of the IMP. This method not only allows adequate dissection of the lymph nodes at the root of the IMA but also protects the left trunk of the IMP. This surgical technique was successfully performed in 45 patients undergoing surgery for colorectal cancer. The bilateral trunks of the IMP were well identified and protected intraoperatively, with no intraoperative complications and satisfactory postoperative urogenital functional outcomes. Therefore, this surgical technique is technically safe and feasible.

In addition, some scholars (16, 17) believe that the integrity of Gerota’s fascia should be maintained to better protect the autonomic nerves below Gerota’s fascia during lymph node dissection. However, we believe that creating an incision in Gerota’s fascia above the junction point of the bilateral trunks of the IMP will facilitate lymph node dissection at the root of the IMA and thus yield better oncological results. Our previous study (24) showed that the rate of no. 253 lymph node metastasis in stage III rectal cancer was 11.0% (29/264). As the **Video** shows, if the integrity of Gerota’s fascia is maintained, it is likely that the dissection of enlarged lymph nodes is not feasible or even omitted. In contrast, incising Gerota’s fascia at a suitable position can better expose the IMP, which may, to some extent, help to achieve better nerve protection.

To the best of our knowledge, this study is the first to report the anatomical relationship between the left trunk of the IMP and the IMA vascular sheath and to propose a novel and reliable nerve-sparing surgical technique for high ligation of the IMA based on anatomical evidence. Due to the limitation of the relatively small sample size, the results need to be validated by further studies with large samples in the near future. Moreover, the oncological outcomes still need to be assessed with long-term follow-up.

## Conclusion

The left trunk of the IMP forms part of the IMA vascular sheath. This novel anatomical evidence-based operative technique for nerve-sparing high ligation of the IMA is technically safe and feasible. However, further validation in larger studies is warranted.

## Data Availability Statement

The datasets generated for this study are available on request to the corresponding authors.

## Ethics Statement

The studies involving human participants were reviewed and approved by The Institutional Review Board of the Fujian Medical University Union Hospital (NO.2020KY092). The patients/participants provided their written informed consent to participate in this study.

## Author Contributions

ZZ and XW conceived of the study, performed the anatomical studies, and prepared the manuscript draft. YH, XL, XZ, and PC critically revised the manuscript for important intellectual content. ZZ and XW performed the data collection and designed the study. All authors contributed to the article and approved the submitted version.

## Funding

This study was supported by the Joint Funds for the Innovation of Science and Technology, Fujian Province (No. 2019Y9101), the Natural Science Foundation of Fujian Province (2020J011030), the Medical Science Research Foundation of Beijing Medical and Health Foundation (B20062DS), and the Medical Innovation Project of Fujian Province (2020CXA025).

## Conflict of Interest

The authors declare that the research was conducted in the absence of any commercial or financial relationships that could be construed as a potential conflict of interest.
